# Occurrence of Antibody-Dependent Enhancement of Avian Infectious Bronchitis in Target Animal Experiments

**DOI:** 10.3390/vetsci13070650

**Published:** 2026-07-02

**Authors:** Lin Cheng, Di Wang, Jia-Rui Zhang, Yi-Han Zhang, Xin-Rui Wu, Ya-Mei Huang, Min Li, Fu-Yan Wang, Yang Zhao, Xin-Feng Han, Min Cui, Yong Huang, Jing Xia

**Affiliations:** 1 College of Veterinary Medicine, Sichuan Agricultural University, Chengdu 611130, China; 202200915@stu.sicau.edu.cn (L.C.); 2543563630@qq.com (D.W.); zjr20020418@163.com (J.-R.Z.); 547589282@qq.com (Y.-H.Z.); 13980214235@163.com (X.-R.W.); 1414177563@qq.com (Y.-M.H.); wangfuyan87@163.com (F.-Y.W.); 18608005730@163.com (Y.Z.); hanxinf@163.com (X.-F.H.); cuimin@sicau.edu.cn (M.C.); 2 Animal Disease Prevention and Control Center of Chengdu City, Chengdu 610000, China; limin057611@163.com; 3 MOE Key Laboratory of Agricultural Bioinformatics, Sichuan Agricultural University, Chengdu 611130, China

**Keywords:** coronavirus, antibody dependent enhancement, complement, infectious bronchitis virus, IBV, ADE

## Abstract

Outbreaks of avian infectious bronchitis virus (IBV) often occur in vaccinated flocks. The antibody-dependent enhancement (ADE) has been proposed as a potential mechanism underlying coronavirus vaccine failure. However, this hypothesis has yet to be substantiated in flocks. This study demonstrates ADE occurrence in IBV (gamacoronavirus) in vitro and in vivo. Using the SPF chicken host model, primary infection with an O-glycosylation-modified attenuated strain enhanced pathogenesis upon secondary homologous/heterologous virulent challenge, increasing morbidity/mortality (≥30%), pathological lesions, and viral loads. Notably, sequentially attenuated infections also induced ADE, suggesting live attenuated vaccine risks. The immune serum raised against the O-glycosylation-modified attenuated strain was also pre-mixed with the challenge strain, and the mixtures were then inoculated into target cells, non-susceptible macrophage cells, or a co-culture of both cell types. The serum-virus complexes replicated poorly in macrophages, yet immune cells amplified the expression of inflammatory factors and ADE-mediated viral replication in target cells, indicating a significant promoting role of immune cells in this process. The concentrations of complement component C3 and neutralizing antibodies in the immune serum were also measured, and results showed that the induction of this ADE is associated with high complement component C3 and low neutralizing antibody titers. These findings highlight risks for vaccines and antibody-based therapeutic strategies of coronavirus infection.

## Simple Summary

When a virus infects a second time, the body’s antibodies can sometimes make the disease worse instead of better. This is called antibody-dependent enhancement. It has been seen in several coronaviruses, but mostly only in laboratory experiments. In this study, we used a common chicken coronavirus (avian infectious bronchitis virus) to ask whether this harmful effect can happen inside a living animal. We first infected chickens with a weakened vaccine-like virus that had been modified by adding sugar molecules (a process called O-glycosylation). When these chickens were later infected with a strong, disease-causing virus, they became much sicker. More than 30% of them died, and they had higher amounts of virus in their bodies. We also found that a blood protein called complement component C3 plays a key role. In the early stage after vaccination, chickens with low levels of protective antibodies but high levels of C3 showed the harmful effect. However, when protective antibodies were high, the effect did not happen. Our findings warn that some live vaccines might accidentally cause severe disease upon later infection. This knowledge helps scientists design safer coronavirus vaccines for animals and possibly for humans.

## 1. Introduction

Antibody-dependent enhancement (ADE) is a phenomenon that exacerbates disease progression following viral reinfection. The occurrence of ADE has been widely demonstrated in numerous viral infections, such as dengue fever [1], ZIKA [1], porcine reproductive and respiratory syndrome [2]. It has also been observed in various coronaviruses, including feline infectious peritonitis virus [3], SARS-CoV-2 [4], SARS-CoV [5], MERS-CoV [6] and porcine epidemic diarrhea virus [7]. Coronavirus ADE mechanisms remain poorly understood, with evidence mainly from in vitro or non-natural host models. Our preliminary studies identified avian infectious bronchitis virus (IBV), a prototypical gammacoronavirus, as a potential ADE-inducing pathogen. Accordingly, this study employs IBV as a model to explore the unique mechanisms of coronavirus ADE.

IBV infects the respiratory, urinary, and reproductive systems of chickens, leading to inflammatory responses, cellular damage, and hemorrhage in the trachea, lungs, kidneys, and oviducts. IBV infections commonly emerge in vaccinated chicken flocks, accompanied by the co-circulation of multiple viral genotypes. This vaccine failure is generally attributed to low neutralizing antibody titers or antigenic divergence between field strains and vaccine strains [8,9]. However, the virus still effectively replicates in the vaccinated chickens by homologous genotype vaccines [10]. The known reasons, such as low neutralizing antibody titers or antigenic differences, cannot fully account for this observation. There remains an unidentified mechanism underlying immunization failure in IBV infection. Clinical studies have confirmed that ADE results in immune failure in both animals and humans, suggesting that ADE may serve as one of the key drivers of IBV-associated immune failure.

ADE refers to a phenomenon in which specific sub-neutralizing antibodies bind to the viruses during pathogen infections, interact with receptors on the surface of immune/target cells, thereby facilitating viral entry into immune cells, resulting in enhanced viral replication and immune activation. These specific sub-neutralizing antibodies may be the antigen epitopes induced specific antibodies [11], complement [11], or anti-glycan antibodies [12]. Enhanced viral endocytosis mediated by Fc receptors (FcRs) represents one of the core mechanisms of canonical ADE. In this process, antibodies attach to FcRs on immune cell surfaces via their Fc domains, facilitating viral entry into cells with low susceptibility. For example, certain sub-neutralizing antibodies against FIPV, SARS-CoV, SARS-CoV-2 and MERS-CoV trigger ADE via this classical FcR pathway [13,14,15]. The C1q complement pathway is recognized as the second major pathway driving ADE. Unlike FcRs, which have a limited tissue distribution, complement receptors are broadly expressed across multiple cell types, including immune cells and IBV target cells. Due to the lack of appropriate research models and the complexity of ADE pathogenesis, direct evidence for complement-mediated ADE in coronaviruses is still lacking. In addition, coronavirus-induced ADE also exhibits non-FcRs-dependent pathways [7,16]. Different ADE mechanisms can be activated by coronaviruses, depending on the functional domains targeted by sub-neutralizing antibodies. This indicates that the mechanisms of coronavirus ADE are associated with antibodies and their binding domains on viral proteins.

Collectively, the diversity of participating antibodies and cell types accounts for the complexity of ADE mechanisms. In previous studies, chickens immunized with the attenuated IBV Sczy3C100^-S1M422T^ strain (with an O-glycosylation mutation) showed a significant increase in viral load after challenge with heterologous virulent IBV strains, suggesting a potential ADE phenomenon [17]. The present study investigates the relationship between viral glycosylation and ADE, aiming to elucidate the causes of vaccine failure and inform the development of safer vaccines.

## 2. Materials and Methods

### 2.1. Viruses, Cells, Chickens and Eggs

Six virulent IBV strains were isolated from infected flocks in China and maintained in our laboratory, comprising four GI-19 genotypes (Sczy3, A/CK/MeiShan/17 [MS], JJ and ZZX), one GI-7 genotype CN, and one GVI-1 genotype ZQX. The attenuated Sczy3C100 strain (abbreviated as C100) was derived from Sczy3 by serial passage (100×) in chicken embryonic kidney (CEK) cells [10]. The Sczy3C100^-S1M422T^ variant (abbreviated as M422T) carries a conserved O-glycosylation site at 422 (Threonine [T]) of the S protein [17]. The GI-1 genotype H120 vaccine strain was obtained from the China Institute of Veterinary Drug Control (Beijing, China). The GI-13 genotype 4/91 vaccine strain was supplied by Intervet International B.V. (Boxmeer, The Netherlands). The GI-19 genotype LDT3-A strain was supplied by Harbin Weike Biotechnology Co. Ltd. (Harbin, China). The chicken macrophage HD11 cell line (non-host cell) was cultured in Dulbecco’s modified Eagle’s medium supplemented with 10% fetal calf serum at 37 °C with 5% CO_2_. The viral host cells CEK were prepared as previously described [18]. Specific pathogen-free (SPF) embryos and SPF chickens were purchased from the Beijing Merial (Beijing, China).

### 2.2. Determination of ADE Induction In Vivo

**Figure 1:**
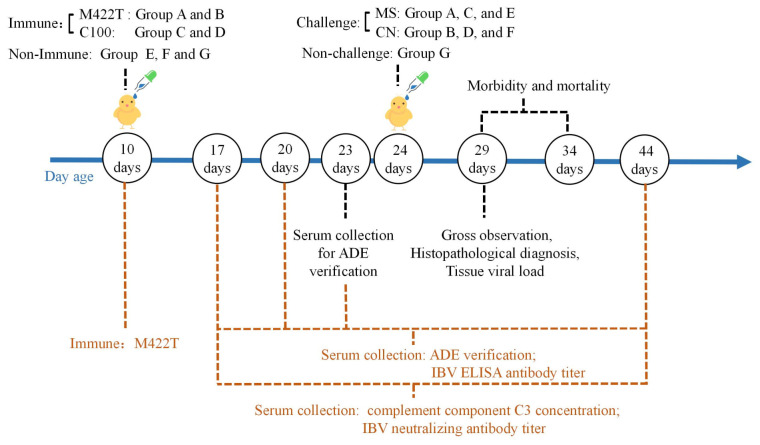
The immune and challenge schedule. The black dashed line represents the timeline of Section 2.2 and Section 2.3; the orange dashed line represents the timeline of Section 2.4. Sczy3C100^-S1M422T^ strain is abbreviated as M422T; the Sczy3C100 strain is abbreviated as C100. Group A is the homologous immune-challenge Sczy3C100^-S1M422T^-MS group. Group B is the heterologous immune-challenge Sczy3C100^-S1M422T^-CN group. Group C is the homologous immune-challenge Sczy3C100–MS control group. Group D is the heterologous immune-challenge Sczy3C100-CN control group. Groups E and F are the challenge control groups, with Group G serving as the control group.

To assess IBV-induced ADE in vivo, we followed an immunization protocol established in our previous work [17]. A total of 70 ten-day-old SPF chickens were randomly divided into seven groups (A–G; n = 10/group) in this research. The immune and challenge schedule is shown in Figure 1. Briefly, Groups A and B were inoculated with Sczy3C100^-S1M422T^; groups C and D were inoculated with the parent strain Sczy3C100; groups E and F served as challenge controls; and group G was administered PBS as a negative control. Immunization was performed via ocular mucosal and intranasal routes with 10^4.5^ TCID_50_/bird. At 14 days post-immunization (d.p.i.), groups A, C, and E were challenged with the homologous GI-19 genotype strain MS, and groups B, D, and F were challenged with the heterologous GI-7 genotype strain CN. Birds were inoculated at a dose of 10^3.9^ TCID_50_ challenge viruses via ocular and intranasal routes. Each group was housed in a separate isolated animal room, and all animal husbandry and care procedures for each room were performed by one dedicated researcher. This design established the standard immunization-challenge protocol, which subsequently served as the model for the other in vivo IBV-ADE study. The pathogenicity of challenge infections was evaluated based on morbidity, mortality, gross observations, histopathological lesions, and viral loads in target organs. All assessments were conducted under a double-blind design.

Morbidity and mortality: Clinical signs were monitored daily for 10 days post-challenge (d.p.c.). Birds presenting whitish diarrhea and respiratory symptoms including coughing, open-mouth breat hing and head shaking were recorded as infected. Respiratory hemorrhage and kidney lesions observed in dead chickens were also defined as signs of infection.

Gross observation: At 5 d.p.c., half of the chickens (four or five chickens) in each group were randomly selected. Macroscopic lesion scores (MLSs) of the trachea and kidneys were graded as follows: 0 for normal, 1 for mild lesion, 2 for serious lesion (Figure S1). Tracheal and kidney tissues from birds that died before 5 d.p.c. were also scored according to the same criteria, and the data were incorporated into the 5 d.p.c. dataset.

Histopathological lesions: parts of the trachea and kidney of four or five chickens in each group were collected at 5 d.p.c. and examined via H&E staining for lesion analysis.

Tissue viral load: At 5 d.p.c., trachea and kidney of four randomly selected birds in each group were collected. Viral load of the diluted supernatants was determined by SYBR Green I-based RT-PCR, as previously described [19]. Each sample was measured in duplicate.

### 2.3. Determination of ADE Induction In Vitro

To assess the universality of ADE, serum samples (n = 20) collected at 13 d.p.i. from Sczy3C100^-S1M422T^-immunized chickens were mixed with 100 TCID_50_/200 µL of MS strain at 37 °C for 1 h. The MS strain and sera of the parent strain Sczy3C100 were set as the virus control and serum + virus control, respectively. These serum-virus complexes were then inoculated onto CEK cells. Following 1 h adsorption, the supernatant was discarded, and cells were washed with PBS. The cultures were collected to measure viral RNA copy number at 48 h post-incubation. Samples exhibiting higher viral RNA levels than the MS challenge control group were designated as ADE-positive.

To determine the optimal serum dilution for ADE in vitro, an ADE-positive serum sample was subjected to two-fold serial dilution. Each dilution was mixed with 100 TCID_50_/200 µL of MS strain for ADE observation in CEK cells, with triplicate wells per dilution. The undiluted positive serum sample was set as the control group, which was directly incubated with CEK cells without adding the virus. The cultures were collected to measure viral RNA copy number at 48 h post-incubation. The dilution with the highest viral load in the serum dilution groups was selected as the optimal dilution. Using this optimal dilution, the number of syncytia in the cells was counted, the TCID_50_ and viral nucleic acid copy number were determined at 48 h post-incubation, and the Sczy3C100 serum + MS group and MS-infected group were set as the control. The viral growth kinetics at this optimal dilution were also assessed by sampling at 12 h intervals (12–72 h post-incubation) for RT-qPCR analysis [19].

To determine whether ADE requires the participation of both immune cells and susceptible cells, five ADE-positive serum samples were tested. The HD11 cells were seeded on coverslips and co-cultured with CEK cells. The Sczy3C100^-S1M422T^ serum + MS virus complexes were incubated with co-culture of HD11 and CEK cells (HD11 + CEK + Serum + Virus group), CEK monocultures (CEK + Serum + Virus), and HD11 monocultures (HD11 + Serum + Virus), respectively. The HD11 + CEK + Virus, CEK + Virus, and HD11 + Virus groups were set as the controls. At 48 h post-incubation, viral RNA copy numbers and TCID_50_ titers were quantified. Additionally, the mRNA expression levels of pro-inflammatory cytokines (IL-1β, IL-6, TNF-α) were analyzed using relative RT-qPCR [20]. Immune complex adsorption and endocytosis patterns were evaluated as previously described [21]. Briefly, the immune complex and 100TCID_50_ of the MS strain were inoculated onto HD11 cells, respectively. Following a 1 h viral attachment period at 4 °C, the cells were washed three times with DMEM and further incubated at 37 °C for 1 h. After an additional three washes with DMEM, the cells were treated three times with an acidic solution (pH 3.0) consisting of 0.1 M glycine and 0.1 M NaCl, adjusted with hydrochloric acid. Samples were collected both after the attachment phase at 4 °C and following the acid wash treatment. Viral RNA copy numbers in these samples were quantified using qRT-PCR.

To determine whether the ADE was complement mediated, we performed complement inactivation assays. The ADE-positive serum was heat inactivated (56 °C, 30 min) prior to ADE in CEK cells. The inactivated serum and non-inactivated ADE-positive serum were separately pre-mixed with 100 TCID_50_/200 µL of the MS strain at 37 °C for 1 h, followed by infection of CEK cells. An equivalent amount of the MS strain alone was set as the control group. Viral nucleic acid copy numbers in each group were determined at 48 h post-infection.

### 2.4. Antibody Analysis of IBV-ADE Induction

Sera were collected at 7, 10, 13, and 20 d.p.i. from chickens immunized with Sczy3C100^-S1M422T^ and mixed with 100 TCID_50_ of the MS strain. The immune complexes were inoculated into CEK cell cultures. Viral RNA copy numbers in the cell and supernatant mixture were quantified via RT-qPCR at 48 h post-incubation.

For the determination of complement component C3 protein concentration, a Sandwich ELISA kits (YJ061662C, C3 ELISA KIT, YUANJU BIO, Shanghai, China) were purchased for detection, and all procedures were performed as per the manufacturer’s instructions. Additionally, an indirect ELISA method established previously in our laboratory was employed to determine the concentration of IBV antibodies. Briefly, the optimal dilutions of antigen (parental strain Sczy3C100 of Sczy3C100^-S1M422T^, 1:20) and serum (1:200) were determined via checkerboard titration. A horseradish peroxidase-conjugated goat anti-chicken secondary antibody was used, and color development was performed with TMD substrate. IBV antibody titers in each immunized group were measured using this established method.

For the determination of IBV neutralizing antibodies, serum samples (including the ADE-positive sample) from five chickens at 7 and 20 d.p.i. were selected for microneutralization assays. The detailed procedure was performed in accordance with a previous study [22]. Briefly, equal volumes of 100 TCID_50_ of the MS strain and serial two-fold dilutions of serum were mixed and kept at 37 °C for 1 h. Next, 0.4 mL of the immunized complexes was transferred to CEK cell cultures in 24-well plates (6 wells for each dilution). The plates were incubated for 72 h, and the 50% end-point neutralizing titers were calculated using the Reed and Muench method.

### 2.5. Determination of Homologous and Heterologous Strain-Induced IBV-ADE

To evaluate whether the observed ADE extends to other IBV strains, we conducted using four homologous (ZZX, JJ, Sczy3 and LDT3-A) and three heterologous strains (ZQX, 4/91 and H120). Serum samples from Sczy3C100^-S1M422T^-immunized chickens were individually mixed with these eight strains. The optimal dilution of serum + 100TCID_50_ virus complexes was inoculated into CEK cells, and viral RNA copy numbers were quantified via RT-qPCR at 48 h post-incubation, with virus-only inoculations serving as controls.

Subsequently, we employed the standardized immunization-challenge protocol to assess ADE in vivo; chickens were immunized with Sczy3C100^-S1M422T^ and challenged at 13 d.p.i. with three commercial vaccine strains (H120, 4/91, LDT3-A). Pathogenicity was evaluated based on indicators, including morbidity, mortality, gross lesions, histopathological changes, and viral load in target tissues.

### 2.6. Statistical Analysis

The animal study involved random allocation/sampling with a small sample size, and the method employed was simple random sampling. If abnormal mortality occurs in animals during in vivo experiments or cell exfoliation is observed in in vitro experiments, such data will be excluded from the analysis. The “abnormal mortality” excluded in this study referred only to accidental deaths (e.g., being trampled or pecked), which were confirmed as unrelated to IBV infection by pathological examination. Deaths directly caused by IBV challenge were not excluded and were included in disease severity assessment. The viral RNA copies were tested using an independent-samples T-test. Lesion scores were analyzed using the Mann–Whitney U test in SPSS 27 software (*p* < 0.05 significant, *p* < 0.01 highly significant, and *p* < 0.001 very highly significant).

## 3. Results

### 3.1. The Induction of ADE by IBV In Vivo

**Figure 2:**
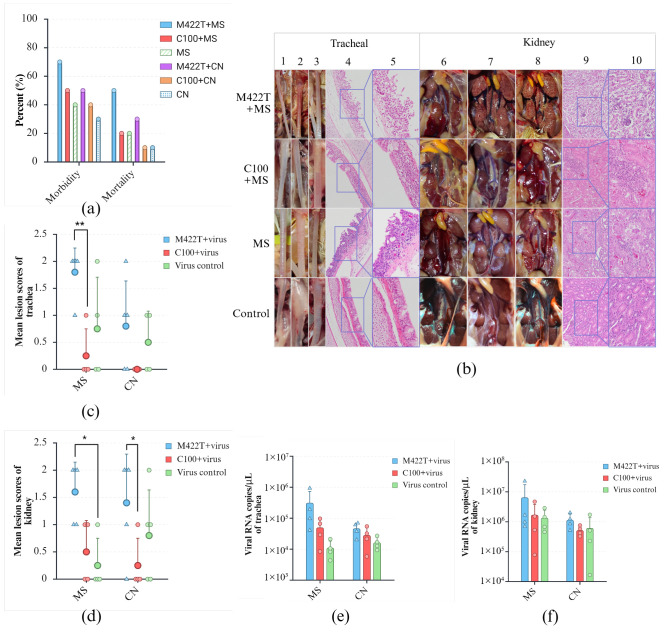
Pathogenic effects following homologous (MS) or heterologous (CN) viral challenge in chickens immunized with the parental IBV strain Sczy3C100 (C100) or its mutant Sczy3C100^−S1M422T^ (M422T). (**a**): morbidity and mortality; (**b**): gross observation and histopathological lesions. The 1–3 represent three tracheal specimens; the 4 (100×) and 5 (200×) were the histopathological sections of the trachea. The 6–8 represent three kidney specimens; the 9 (100×) and 10 (200×) were the histopathological sections of the kidney; (**c**): mean lesion scores of trachea; (**d**): mean lesion scores of kidney; (**e**): Viral load of trachea; (**f**): Viral load of kidney. *: *p* < 0.05, **: *p* < 0.01, the unlabeled group showed no significant difference. Created in Biorender, Jing Xia (2026).

Regarding the pathogenicity of the homologous MS strain in different groups, chickens in the Sczy3C100^-S1M422T^-MS group (Immunization strain: Sczy3C100^-S1M422T^ with an M422T O-glycosylated mutation of the Sczy3C100 attenuated strain, challenged homologous strain: MS) showed the earliest symptom onset (2 days), as well as the highest morbidity (70%) and mortality (50%). These rates were notably higher than those in the Sczy3C100-MS immunized-challenged control group (morbidity: 50%; mortality: 20%) and the MS challenge-only control group (morbidity: 40%; mortality: 20%) (Figure 2a).

The most severe pathological lesions were also observed in the Sczy3C100^-S1M422T^-MS group. The tracheal mucosa was characterized by abundant mucous exudates and diffuse hemorrhage; the histological lesions were characterized by cilia loss, epithelial cell shedding, congestion, hemorrhage, and inflammatory cell infiltration. The kidneys showed significant swelling, accompanied by pallor of the ureters and urate deposits. Histological lesions in the kidney consisted of epithelial cell degeneration, necrosis and shedding, renal tubular exudation, and inflammatory cell infiltration (Figure 2b).

We quantified the gross pathological lesions using the MLSs scores, where higher scores indicate more severe damage. As showed in Figure 2c,d, both tracheal and kidney MLSs of infected chickens in Sczy3C100^-S1M422T^-MS group were higher than in the Sczy3C100-MS immunized—challenged and MS-only control groups (MLSs of trachea: p_M422T-MS_ vs. _C100-MS_ < 0.01; kidney: p_M422T-MS_ vs. _MS_ < 0.05, p_M422T-CN_ vs. _C100-CN_ < 0.05).

Viral loads in the trachea and kidneys of chickens in each group were also measured. Consistent with the trends in morbidity, mortality, and pathological lesions, the viral nucleic acid copy numbers in the trachea and kidneys of chickens in the Sczy3C100^-S1M422T^-MS group were higher than those of chickens in the MS-only challenge group (Figure 2e,f).

A similar pattern was observed for the heterologous challenge strain CN. Chickens in the Sczy3C100^-S1M422T^-CN immunized—challenged group showed higher morbidity and mortality (Figure 2a), more severe pathological lesions (Figure 2b), higher MLS values (Figure 1c,d), and higher viral loads (Figure 2e,f) than those in the Sczy3C100-CN and CN-only control groups.

When comparing the immune protection efficacy of Sczy3C100^-S1M422T^ and its parental Sczy3C100 strain, the parental Sczy3C100 strain also provided incomplete immune protection, as evidenced by higher morbidity and viral loads in target organs than challenge controls, though associated pathology was relatively mild (Figure 2a). Although the morbidity in chickens of the Sczy3C100-immunized-challenge groups was slightly higher than that in the challenge control groups, no significant enhancement in the pathogenicity of secondary infection was observed in terms of mortality and pathogenic observation. This discrepancy may be attributed to data deviations during clinical symptom observation.

In summary, these results indicate that the Sczy3C100^-S1M422T^ attenuated strain not only fails to confer effective immune protection against homologous or heterologous challenge, but also significantly enhances pathogenicity and viral replication in target tissues.

### 3.2. The Induction of ADE by IBV In Vitro

**Figure 3:**
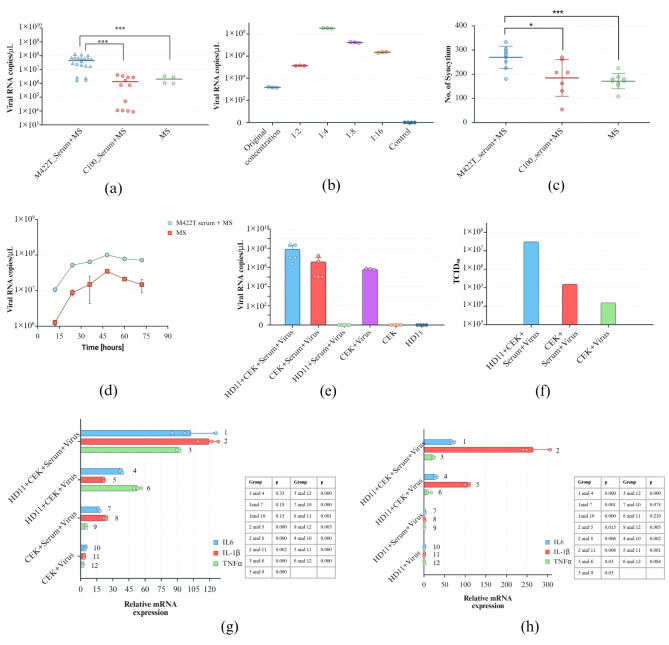
The ADE in vitro. Sczy3C100^-S1M422T^ strain was abbreviated as M422T; the Sczy3C100 strain was abbreviated as C100; MS was the homologous strain. (**a**): Copy number of viral RNA in CEK with serum-viral complexes treated; sample No. M422T serum = 20, C100 serum = 12, MS = 4; (**b**): optimal titer of serum dilution; (**c**): Number of syncytia in CEK cells with serum-viral complexes treated; (**d**): Growth curve of IBV under serum interference; (**e**): Copy number of viral RNA in co-culture of target cells CEK and immune cells HD11; (**f**): Viral TCID_50_ titer in co-culture of target cells CEK and immune cells HD11; (**g**): Expression levels of inflammatory factors in CEK cells co-cultured with HD11; sample No. = 3; (**h**): Expression levels of inflammatory factors in HD11 cells co-cultured with CEK, sample No. = 3. The numbers in g and h were the group code numbers, and the table showed the results of the statistical analyses. *: *p* < 0.05, ***: *p* < 0.001, the unlabeled group showed no significant difference. Created in Biorender, Jing Xia (2026).

A total of 20 Sczy3C100^-S1M422T^ serum samples at 13 d.p.i. were evaluated in host CEK cells. As shown in Figure 3a, the Sczy3C100^-S1M422T^ serum elicited a highly statistically significant elevation of viral load in CEK cells, as compared with the MS group and the Sczy3C100 serum + MS group (*p* < 0.001). In all the test Sczy3C100^-S1M422T^ serum samples, 80% (16/20) of the serum samples were ADE-positive. In the optimal serum dilution assays, all diluted and undiluted positive sera enhanced viral replication, with the 1:4 dilution yielding the highest viral RNA load. The undiluted positive serum control without MS virus tested negative by RT-qPCR (Figure 3b). At 48 h post-inoculation, the MS strain manifested the most robust replication capability in the Sczy3C100^-S1M422T^ serum + MS virus group. The RNA copy number was approximately 100-fold higher than that in the MS strain group. The cytopathic effect was the most pronounced, the number of syncytia (Figure 3c, *p* < 0.001) and TCID_50_ (Sczy3C100^-S1M422T^ serum + MS group: 10^−7.51^/mL, Sczy3C100 serum + MS group: 10^−5.0^/mL, MS strain group: 10^−4.22^/mL) were also the greatest in Sczy3C100^-S1M422T^ serum + MS group. To determine whether the Sczy3C100^-S1M422T^ serum could affect the viral replication kinetics, the growth kinetics of the virus in the Sczy3C100^-S1M422T^ serum + MS group were detected. The results showed that the Sczy3C100^-S1M422T^ serum only increased the viral titers at various time points but did not alter the viral replication kinetics (Figure 3d). Conversely, the immune serum of Sczy3C100 failed to significantly induce the ADE phenomenon in vitro.

These results strongly suggest that the immune serum of Sczy3C100^-S1M422T^ not only lacks neutralizing activity against the homologous strain MS, but also enhances the replication and pathogenicity of IBV in target CEK cells. ADE is defined as a phenomenon in which host antibodies increase viral infection. In this study, immunization with the parental strain Sczy3C100 slightly increased the morbidity and viral load following challenge infection. However, it did not exacerbate the pathological damage in the host, nor did the corresponding immune serum enhance viral replication in vitro. We propose that definitive ADE should be characterized by enhanced infectivity both in vivo and in vitro. Immunization with the mutant met this criterion, demonstrating its capacity to induce robust ADE in the host.

Immune cells often play a critical role in ADE. To investigate whether immune cells modulate the ADE observed in this study, HD11 cells were added to the host CEK cells for ADE assessment. Upon adding HD11 to CEK cells (HD11 + CEK + Serum + Virus), the viral RNA copy number was higher (Figure 3e), the cytopathic effect was intensified (Figure S2), and the viral titer increased (Figure 3f) compared to that in the CEK + Serum + Virus and CEK + Virus groups. We measured the mRNA expression of cytokines in CEK and HD11 cells across the experimental groups, respectively. The results showed that in all groups—whether serum or HD11 cells were added or not—CEK cells infected with MS virus exhibited upregulation of the inflammatory factors IL-6, IL-1β, and TNFα. Among them, the HD11 + CEK + Serum + Virus group showed the highest expression (*p* < 0.01 or <0.001), with an increase of more than 90-fold (Figure 3g). Regarding the inflammatory factor levels in HD11 cells across the groups, upregulation was only observed in the presence of CEK host cells. Consistent with the findings in CEK cells, the HD11 cells in the HD11 + CEK + Serum + Virus group also displayed the most pronounced upregulation in inflammatory cytokine mRNA expression, with IL-1β levels increased more than 250-fold (Figure 3h, *p* < 0.01 or <0.001). These results demonstrate that immune cells can potentiate the ADE response in this system. To eliminate the interference of IBV utilizing HD11 as a potential host cell, experiments were also conducted regarding the RNA copy number, attachment, and endocytosis processes during IBV infection of HD11. The results indicated no signs of viral infection in HD11 cells.

### 3.3. Determination of the Effect of Serum Inactivation on ADE

**Figure 4:**
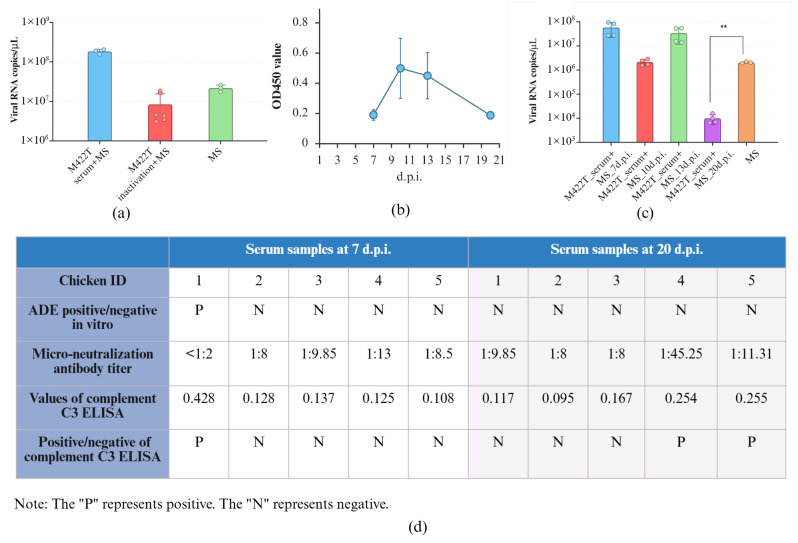
Effect of complement C3 and antiviral antibody concentrations on ADE; (**a**): Effect of serum inactivation on ADE; (**b**): Viral ELISA antibody concentration after immunization with Sczy3C100^-S1M422T^ strain; (**c**): Differences in ADE-induction ability of serum at different d.p.i.; (**d**): Correlation among complement C3 concentration, neutralizing antibody titer, and ADE. **: *p* < 0.01; unlabeled group showed no significant difference. Created in Biorender, Jing Xia (2026).

To determine whether the IBV-ADE was instigated by the complement present in the serum, the serum of Sczy3C100^-S1M422T^ was inactivated at 56 °C for 30 min to inactivate the complement. We observed that the non-inactivated serum significantly enhanced viral replication in CEK cells, whereas heat-inactivated serum exhibited no enhancing effect. Instead, the addition of inactivated serum markedly attenuated viral proliferation (Figure 4a). This phenomenon may be attributed to the effective neutralization of viruses by specific neutralizing antibodies. Therefore, it can be speculated that the ADE observed in this study is associated with complement activity.

### 3.4. Antibody Analysis of IBV-ADE Induction

Serum samples collected at 7, 10, 13, and 20 d.p.i. from chickens immunized with the Sczy3C100^-S1M422T^ strain were tested for IBV antibodies by ELISA. All tested samples were positive with varying antibody concentrations: titers were low at 7 d.p.i., peaked at 10 d.p.i., began to decline at 13 d.p.i., and returned to the 7 d.p.i. level at 20 d.p.i. (Figure 4b).

To assess the ability of sera collected at these time points to induce ADE in vitro, the same serum samples were incubated with 100 TCID_50_ of the MS strain, and co-incubated with CEK cells. Viral loads were quantified at 48 h post-incubation. The mean viral loads at 7, 10, 13 d.p.i. were higher than those in the MS control group, with the highest value at 7 d.p.i., followed by 13 d.p.i., and the lowest at 10 d.p.i. In contrast, the viral loads at 20 d.p.i. were lower than those in the MS control group (*p* < 0.01) (Figure 4c). This indicated that the serum collected at 20 d.p.i. exhibited no enhancing effect on viral replication.

We further selected serum collected at 7 and 20 d.p.i. from five Sczy3C100^-S1M422T^-immuned chickens (including ADE-positive and ADE-negative chickens) to measure complement C3 and neutralizing antibody levels. Chicken No. 1 tested positive for ADE at 7 d.p.i., with very low serum neutralizing antibody titers but high complement component C3 titers (positive). At 20 d.p.i., its serum lost ADE effects; instead, it exhibited high neutralizing antibody titers and low complement component C3 titers (negative). For the other chickens tested (Nos. 2–5), no ADE effect was detected at either 7 or 20 d.p.i. They maintained high neutralizing antibodies and low C3 at 7 d.p.i.; even with elevated C3 in some individuals at 20 d.p.i., robust neutralizing antibodies still prevented ADE (Figure 4d). Collectively, ADE arises under low-neutralizing antibody and high-C3 conditions, while sufficient neutralizing antibodies block ADE regardless of C3 abundance. Notably, this conclusion remains preliminary due to the limited sample size of this cohort.

### 3.5. Homologous and Heterologous Strain-Induced IBV-ADE

**Figure 5:**
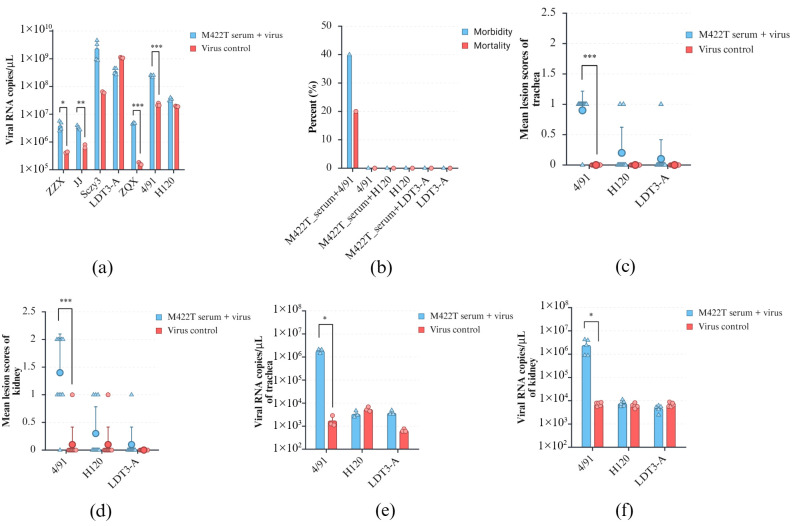
Induction of ADE in vitro and in vivo upon secondary infection with homologous or heterologous strains. Sczy3C100^-S1M422T^ was abbreviated as M422T; (**a**): Copy number of viral RNA in CEK with serum-viral complexes treated. Homologous strains: ZZX, JJ, Sczy3 and LDT3-A; heterologous strains: ZQX, 4/91 and H120; (**b**): morbidity and mortality; (**c**): mean lesion scores of trachea at 5 d.p.c.; (**d**): mean lesion scores of kidney at 5 d.p.c.; (**e**): viral load of trachea sampled at 5 d.p.c.; (**f**): Viral load of kidney sampled at 5 d.p.c. *: *p* < 0.05, **: *p* < 0.01, ***: *p* < 0.001, unlabeled group showed no significant difference. Created in Biorender, Jing Xia (2026).

Serum from Sczy3C100^-S1M422T^-immunized chickens was mixed separately with four homologous GI-19 genotype strains (Sczy3, JJ, ZZX, LDT3) and three heterologous different genotype strains (ZQX, 4/91, H120); these serum-viral complexes were subsequently employed to infect CEK to observe ADE. The results demonstrated that three homologous strains (Sczy3, JJ, ZZX) and two heterologous strains (ZQX and 4/91) manifested varying levels of ADE and significantly enhanced viral replication in the cells (*p* < 0.05 or *p* < 0.001) (Figure 5a). These homologous and heterologous challenge tests suggest that ADE is mediated by serum from Sczy3C100^-S1M422T^-immunized chickens, not by the challenge strains themselves.

It is worth noting that among the ADE-positive cell groups, 4/91 is a commercial attenuated vaccine strain, while all the other strains are virulent. We further verified whether ADE could occur during infection with attenuated strains in vivo. Results showed that birds immunized with Sczy3C100^-S1M422T^ and subsequently infected with 4/91 strain exhibited 40% morbidity and 20% mortality (Figure 5b), conspicuous damage to the trachea and kidneys (*p* < 0.001) (Figure 5c,d), accompanied by severe inflammation, hemorrhage, and cell necrosis (Figure S3), and a significant higher viral load in target tissues (*p* < 0.05) (Figure 5e,f). Conversely, in the challenge control group inoculated solely with 4/91, no clinical signs or deaths were observed, and no obvious pathological changes were detected. No ADE was detected in the other two vaccine strains, H120 and LDT3-A. These results indicated that an attenuated strain could also exhibit strong pathogenicity when it infected immune chickens and triggered the ADE phenomenon.

### 3.6. Distribution of the Glycosylation Sites

**Figure 6:**
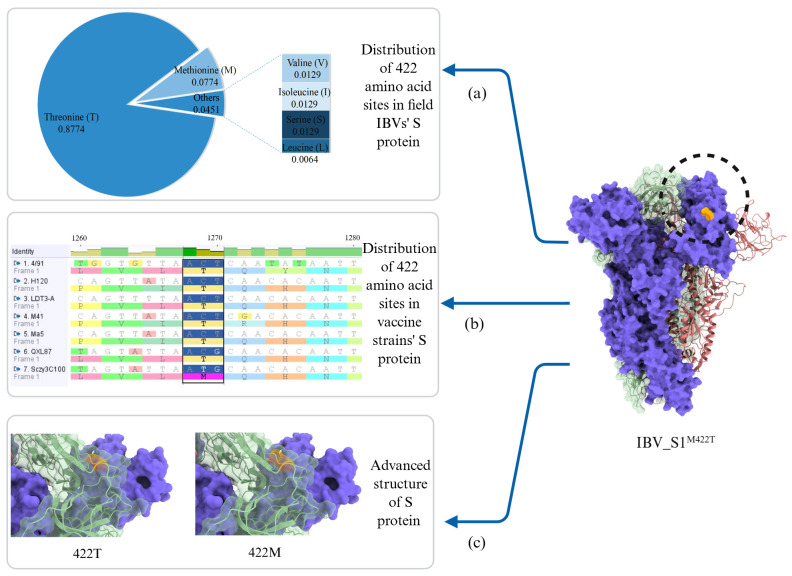
Structural prediction of O-glycosylation modification at amino acid position 422 (422T) in the S protein and its distribution in IBVs; (**a**): structural prediction of S protein, the yellow mark represents the 422 amino acid site; (**b**): distribution of 422T in field IBVs; (**c**): distribution of 422T in IBV vaccine strains, the 422 amino acid position is marked with the box. Created in Biorender, Jing Xia (2026).

We also investigated the distribution of the M422T mutation. Analysis of 195 recent (past five years) isolates of IBV demonstrated that the O-glycosylated threonine (T) at position 422 in the S1 subunit was highly prevalent, occurring in 87.74% of circulating strains (Figure 6a). Notably, all six widely used commercial IBV vaccine strains also carried this O-glycosylated threonine (Figure 6b), implying that current vaccines may carry a potential risk of inducing ADE. Notably, although the 422T residue is commonly found in commercial vaccine strains, its presence warrants further empirical investigation rather than being interpreted as direct evidence of ADE risk. This O-glycosylation modification didn’t alter the viral surface structure (Figure 6c).

## 4. Discussion

The pathogenic impact of ADE in coronavirus infections remains poorly understood. Limited clinical evidence and complex underlying mechanisms hinder the verification of ADE effects in susceptible populations. This study provides definitive evidence of ADE occurrence in IBV infection, demonstrating its effects both in vitro and in vivo. IBV infection causes up to 30% mortality in field cases and 80% in experimental infections [23,24,25]. Despite widespread vaccination, it is still predominantly in China [26,27]. Apart from low neutralizing antibody levels or antigenic differences, our findings reveal that ADE represents an alternative mechanism contributing to vaccine failure. ADE increased morbidity and mortality rates by 30%, underscoring its significant risk to susceptible hosts. We also observed that sequential infection with two attenuated IBV strains exhibited ADE-induced clinical outcomes resembling virulent infection, and characteristic lesions such as upper respiratory tract pathology and interstitial nephritis. The high prevalence of the 422T site in both circulating strains and vaccine strains indicates a potential risk of ADE in clinical settings. Notably, the correlation between the 422T residue and ADE occurrence observed in our experimental system does not automatically translate to vaccine-elicited ADE risk in the poultry industry. ADE is influenced by multiple factors including vaccine strain backbone, immunization dose, post-vaccination time points, neutralization antibody titer, complement status, challenge strain characteristics, host genetic background, and so on.

We further investigated the underlying mechanism of this ADE. In vitro studies demonstrated that immune serum directly enhanced viral replication in target CEK cells. Notably, this ADE was not dependent on a reaction. The addition of immune cells into CEK cells could activate macrophages. The macrophage activation drives pathological cytokine storm responses, resulting in systemic impairment of antiviral defenses and potentiation of viral replication. These observations indicate that the initiation and progression of ADE in vivo involve interactions among multiple cell types. The FcγRs have been implicated in coronavirus ADE in most studies [11,28]. Their restricted expression on immune cells (e.g., macrophages, B cells, and natural killer cells) limits their role in infections targeting epithelial cells, such as those caused by IBV and SARS-CoV-2. Instead, our data suggest that the complement pathway may play a critical role in ADE. This study found that the occurrence of IBV-ADE was not determined solely by the level of neutralizing antibodies, but exhibits a synergistic relationship with both neutralizing antibody concentration and complement C3 levels. Under conditions of low neutralizing antibody levels and high complement C3 concentrations, immune serum significantly enhanced IBV replication in CEK cells. However, as neutralizing antibody levels increased, the phenomenon of enhanced infection was no longer observed. Some researchers have also shown that the complement system exerts a marked bidirectional modulation of antibody function: it can significantly enhance the neutralizing titers of certain antibodies, while also enabling non-neutralizing antibodies to acquire infection-enhancing capacity. This bidirectional regulation depends on antibody concentration, antibody type, and epitope specificity [29]. This mechanism provides a more plausible explanation for ADE-mediated infection of respiratory epithelial cells and expands our understanding of coronavirus pathogenesis [11].

We investigated the viral structural components responsible for this ADE. The antigenic differences are the popular explanation. A previous study had analyzed the antigenic characteristics of strains used in this study; the homologous IBVs belonged to the same serotype with a high probability [30], the M422T mutation has no effect on the antigenicity of the parent strain SczyC100 [17]. The heterologous strain (4/91) was antigenically distinct from Sczy3C100, showing significant serological differences [22]. These results indicate the observed ADE was not correlated with the antigenic relatedness between the primary and secondary infectious strains, as both closely related and highly divergent antigens could trigger ADE. Comparison between the Sczy3C100^-S1M422T^ and its parental strain revealed that Sczy3C100^-S1M422T^ immune serum induced significantly enhanced viral pathogenicity and replication capacity. The Sczy3C100^-S1M422T^ mutant contains a point mutation at position 422 in the S1 subunit, which introduces an O-glycosylation modification without markedly altering viral surface structure or antigenicity. Our previous studies have systematically characterized glycosylation patterns of IBV spike protein. The O-glycosylation modification sites of S protein had sialylated and fucosylated glycans as side chains with distinct structural features [17]. We consider that the glycan chains of O-glycosylation modification in Sczy3C100^-S1M422T^ may excessively activate complement-mediated signaling cascades. This exaggerated immune response leads to tissue damage and disease exacerbation. The severity of this response remains unaffected by the antigenic differences between sequentially infecting strains.

Based on these findings, we proposed the ADE-induction process. The proposed mechanism may involve the following steps: During the primary infection, the O-glycosylated side chains of the IBV S protein elicit the production of antibodies and complement component C3 in chickens. Upon secondary infection, antibodies and complement component C3 bind to virus particles, forming virus-antibody-complement complexes. The complexes then interact with complement receptors on target cell surfaces, facilitating viral entry and enhancing intracellular replication. Concurrently, engagement of receptors on inflammatory cells initiates a robust inflammatory cascade, culminating in a cytokine storm. The massive release of pro-inflammatory mediators leads to widespread damage to both immune cells and target tissues, while simultaneously suppressing antiviral responses. Consequently, the compromised immune system fails to efficiently eliminate infected cells, thereby creating a permissive environment for enhanced viral replication in target tissues. This cascade ultimately results in the observed exacerbation of pathogenicity in the infected strains. Some steps in this hypothetical mechanism lack more precise validation—for instance, whether complement component C3 is involved, how the complement component C3 complex enters CEK cells, and how it activates immune cells. These issues require further rigorous demonstration. In addition, due to the limited sample size, the relationship between complement C3, neutralizing antibodies, and ADE requires further validation in subsequent studies with expanded sample numbers.

## 5. Conclusions

This study demonstrated that ADE can occur in IBV-immunized chickens, leading to vaccine failure. Upon the occurrence of ADE in infected chickens, the vaccine strain administered during primary immunization not only fails to confer effective immune protection but also significantly enhances the pathogenicity of subsequently infecting viral strains. This ADE phenomenon is primarily induced by glycosylation modifications of the S1 subunit in the IBV attenuated vaccine strain used for initial immunization, with the participation of immune cells further potentiating the enhancement. This ADE process may be induced by complement component C3. Notably, sequential attenuated infections also induced ADE, suggesting live vaccine risks. These findings highlight risks for vaccines and antibody-based therapeutic strategies of coronavirus infection.

## Data Availability

The original contributions presented in this study are included in the article. Further inquiries can be directed to the corresponding authors.

## Supplementary Materials

The following supporting information can be downloaded at: https://www.mdpi.com/article/10.3390/vetsci13070650/s1, Figure S1. The scoring criteria of gross lesions in the trachea and kidney of IBV-infected chickens; Figure S2. In the IBV ADE effect model in vitro, the growth status of CEK cells at 48 h post-incubation, and the black arrows were syncytia; Figure S3. The gross observation and histopathological lesions of chickens that were immunized with the Sczy3C100^-S1M422T^ attenuated strain and challenged with three commercial attenuated vaccine strains (4/91, H120 and LDT3-A).

## Abbreviations

The following abbreviations are used in this manuscript:

ADE	Antibody-dependent enhancement
IBV	Avian infectious bronchitis virus
FcRs	Fc receptors
Sczy3C100^-S1M422T^	M422T
Sczy3C100	C100
d.p.i.	Days post-immunization
d.p.c.	Days post-challenge
MLSs	Macroscopic lesion scores
CEK	Chicken embryo kidney
SPF	Specific pathogen-free
