# Supplementary material for: Occurrence of Antibody-Dependent Enhancement of Avian Infectious Bronchitis in Target Animal Experiments
Source: Vet Sci. 2026 Jul 2;13(7):650. doi: 10.3390/vetsci13070650 (PMC13417425; doi:10.3390/vetsci13070650)
Supplement: Supplementary file 1 [file vetsci-13-00650-s001.zip › vetsci-4395634-supplementary.pdf]

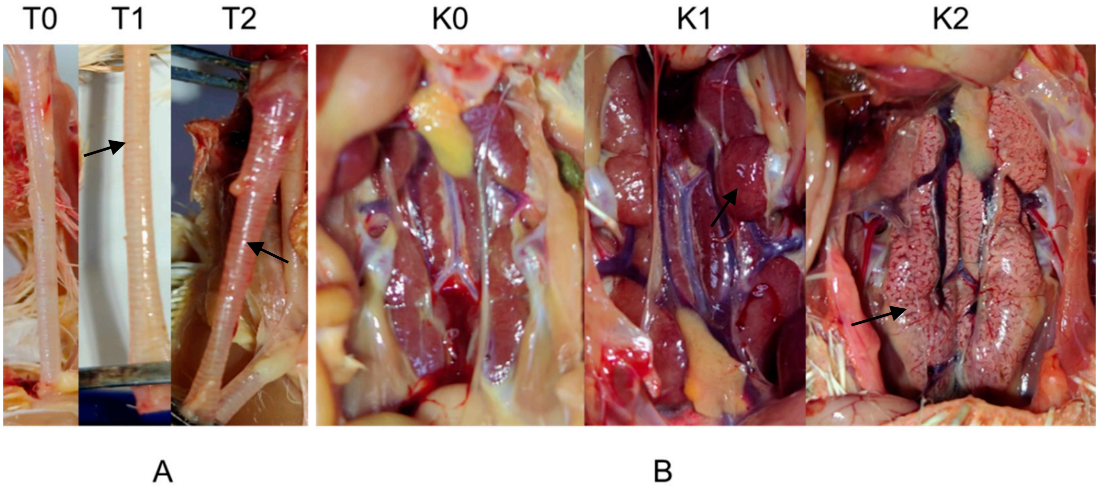

Figure S1. The scoring criteria of gross lesions in trachea and kidney of IBV infected chickens. A: hemorrhage in the trachea (black arrow). Tracheal lesions were scored as follows: 0 (T0) for normal; 1 (T1) for mild haemorrhage; 2 (T2) for diffuse severe haemorrhage. B: kidney enlarged significantly with pallor of ureters that contain urate deposits (black arrow); 0 (K0) for normal; 1 (K1) for mild enlargement; 2 (K2) for severe enlargement or urate deposition.

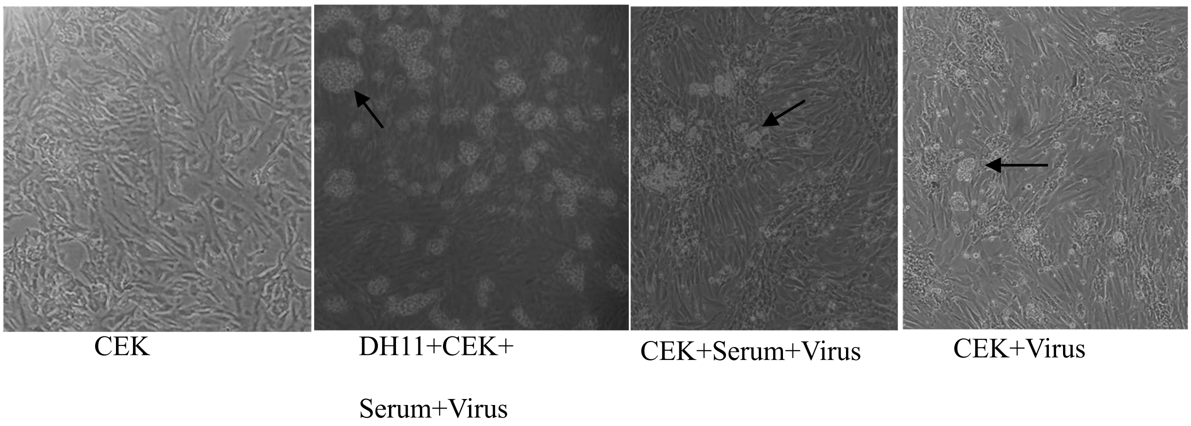

Figure S2. In the IBV ADE effect model in vitro, the growth status of CEK cells at 48 h post incubation, and the black arrows were syncytia.

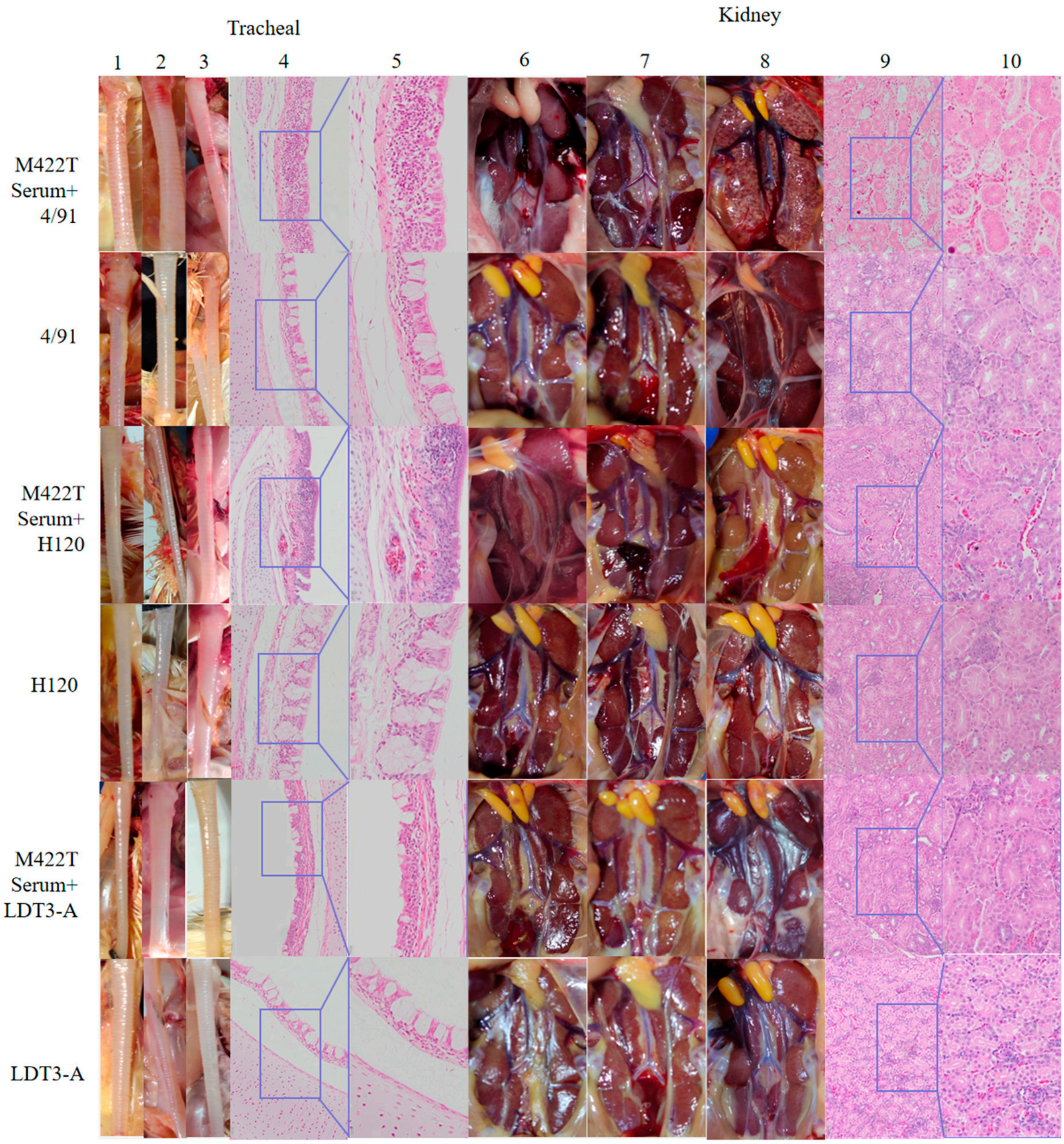

Figure S3. The gross observation and histopathological lesions of chickens which were immunized with the Sczy3C100^{M422T} attenuated strain and challenged with three commercial attenuated vaccine strain (4/91, H120 and LDT3-A). The trachea and kidneys were also sampled at 5 days post-challenge. The 1－3 represent three tracheal specimens, the 4 (100×) and 5 (200×) were the histopathological sections of the trachea. The 6－8 represent three kidney specimens, 9 (100×) and 10 (200×) were the histopathological sections of the kidney. Only the 4/91-challenged group immunized with Sczy3C100^{M422T} （M422T Serum + 4/91) showed significant gross lesions (tracheal hemorrhage, renal swelling, urate deposition) and severe histological damage. The M422T Serum + H120 group exhibited mild hemorrhage and inflammatory infiltration. No obvious pathological features were observed in other groups.
